# Maximal use of 0.05% topical isotretinoin in patients with congenital ichthyosis results in low systemic exposure

**DOI:** 10.1002/bcp.70423

**Published:** 2025-12-17

**Authors:** Holm Schneider, Christopher G. Bunick, Kathrin Hillmann, Thy N. Huynh, Steven Kempers, Nicolai Peschel, Ulrike Blume‐Peytavi, Joyce M. C. Teng, Alan M. Mendelsohn, John Stinson, Lara Wine Lee

**Affiliations:** ^1^ Department of Pediatrics University Hospital Erlangen Erlangen Germany; ^2^ Department of Dermatology Yale School of Medicine New Haven Connecticut USA; ^3^ Department of Dermatology, Venereology and Allergology Charité Universitätsmedizin Berlin Berlin Germany; ^4^ Department of Dermatology University of Mississippi Jackson Mississippi USA; ^5^ Associated Skin Care Specialists New Brighton Minnesota USA; ^6^ Department of Dermatology Stanford University School of Medicine Palo Alto California USA; ^7^ Timber Pharmaceuticals, LEO Pharma Company Madison New Jersey USA; ^8^ LEO Pharma A/S Ballerup Denmark; ^9^ Department of Dermatology Medical University of South Carolina Charleston South Carolina USA

**Keywords:** ARCI, pharmacokinetics, retinoids, RXLI, TMB‐001

## Abstract

Congenital ichthyoses (CI) are rare, inherited skin disorders characterized by hyperkeratosis, scaling and fissuring that significantly impair patients' quality of life. Treatment options are limited, with systemic retinoids reserved for severe cases owing to their adverse effect profile. This open‐label, single‐arm, maximal‐use trial investigated the systemic exposure and safety of a topically administered isotretinoin ointment (TMB‐001 0.05%) in patients with moderate‐to‐severe CI. Thirty‐four patients aged ≥6 years applied TMB‐001 0.05% under maximal‐use conditions (twice daily to 75%–90% of the body surface area) for 14 days, with continued application for another 10 weeks. Exposure levels for TMB‐001 0.05% and its metabolites were < 1% of those observed after single oral administration of 80 mg isotretinoin to healthy adults. A majority of patients had local safety or tolerability issues, most of which were mild. Overall, the treatment was well tolerated with no evidence of systemic toxicity.

**What is already known about this subject**

- Systemic retinoids, including oral isotretinoin, have shown efficacy in the treatment of congenital ichthyosis (CI) but may be associated with significant adverse effects.
- A proprietary, topical, polyethylene glycol‐based isotretinoin ointment (TMB‐001 0.05%) has been formulated as a potentially effective and safer alternative to oral isotretinoin for treatment of CI.

**What this study adds**

- Fourteen‐day topical application of TMB‐001 0.05% in CI patients resulted in systemic exposure levels < 1% of those following a single oral dose of isotretinoin.
- Overall, TMB‐001 0.05% was well tolerated in this maximal‐use trial.
- The results may inform further investigation of isotretinoin formulations for topical treatment of various skin conditions.

## 1 INTRODUCTION

Congenital ichthyoses (CI) are a heterogeneous group of inherited skin disorders characterized by abnormal epidermal differentiation and skin barrier impairment. In autosomal recessive CI (ARCI; prevalence 1:100 000) and recessive X‐linked ichthyosis (RXLI; prevalence 1:3000 males), main features are widespread hyperkeratosis and scaling of the skin, typically accompanied by painful fissuring, pruritus and major impact on quality of life.^1^, ^2^ Management of CI is typically limited to the use of emollients, keratolytic agents and in severe cases systemic retinoids (vitamin A analogues).^1^, ^3^ Investigational use of systemic retinoids, such as oral isotretinoin, has shown efficacy in the treatment of CI but may be associated with significant adverse effects, including potential teratogenicity as well as skeletal, blood and hepatic toxicity.^3^, ^4^ Hence, any repeated administration of systemic retinoids requires careful monitoring.

A proprietary, topical, polyethylene glycol‐based isotretinoin ointment (TMB‐001 0.05%) has been formulated as a potentially effective and safer alternative to oral isotretinoin for the treatment of CI.^5^ TMB‐001 0.05% was investigated in the recent Phase 3 ASCEND trial (NCT05295732). Here, we report the systemic exposure to TMB‐001 0.05% and its metabolites during maximal use in a subset of ASCEND trial participants, thereby adding to the limited data published on the pharmacokinetics (PK) of topical isotretinoin, particularly in children and adolescents. Furthermore, we compare these data with exposure data obtained after single oral administration of 80 mg isotretinoin to healthy adults.

## 2 METHODS

Data were obtained from an open‐label, single‐arm, maximal‐use part of the Phase 3 multi‐centre trial ASCEND (hereafter referred to as ASCEND‐MUsT), investigating the PK of topically applied TMB‐001 0.05% in patients aged ≥ 6 years with moderate‐to‐severe, genetically confirmed RXLI or ARCI. Eligibility criteria are described online (https://clinicaltrials.gov/study/NCT05295732). For PK assessment, TMB‐001 0.05% was dosed for 14 days under conditions of maximal use, defined as twice‐daily application to 75%–90% of the body surface area (BSA). For safety and exploratory efficacy assessments, the application was continued for another 10 weeks. The approximate maximum daily dose of isotretinoin, based on twice‐daily application to 90% of the BSA, was estimated to be 0.034 g/day in an average adult male and 0.013 g/day in an average 6‐year‐old.

Additionally, data were obtained from an open‐label, single‐arm, single‐dose Phase 1 monocentric trial (protocol no. C1D02307/TMB01‐101) investigating the PK of orally administered isotretinoin (Claravis™, 2 × 40 mg capsules) in healthy adult males. Claravis™ is an FDA‐designated reference standard for the reference‐listed drug (Accutane®, unavailable in the US since 2009).

Both clinical trials were approved by an IRB/IEC at each centre and conducted in accordance with the Declaration of Helsinki principles and Good Clinical Practice guideline. All participants gave their written informed consent. Parents or legal guardians provided written informed assent for participants < 18 years of age.

PK analysis was based on intensive sampling from patients aged ≥12 years in ASCEND‐MUsT and from participants in the oral‐dose trial and sparse sampling from patients aged 6–11 years in ASCEND‐MUsT (Tables S1–S2). Plasma concentrations of isotretinoin, its interconvertible geometric isomer tretinoin and their main metabolites, 4‐oxo‐isotretinoin and 4‐oxo‐tretinoin, were detected using high‐performance liquid chromatography–tandem mass spectrometry. PK parameters were derived from plasma concentration–time course data using non‐compartmental analysis and the linear‐up/log‐down method. Plasma concentrations and derived PK parameters were summarized by age group using descriptive statistics, excluding concentrations that were missing or below the limit of quantification.

Safety and tolerability of TMB‐001 0.05% after topical application for 12 weeks in ASCEND‐MUsT were assessed based on reported adverse events (AEs) and local skin reactions (LSRs; burning/stinging, oedema, erosions, erythem, and suspected allergic contact dermatitis). Efficacy was evaluated using the Investigator's Global Assessment (IGA; Table S3).

The key ligand in this article is hyperlinked to the corresponding entry online (http://www.guidetopharmacology.org) and is permanently archived in the Concise Guide to PHARMACOLOGY 2023/24.^6^

## 3 RESULTS AND DISCUSSION

ASCEND‐MUsT enrolled 34 patients (52.9% males; 76.5% ARCI, 23.5% RXLI; age range: 4–64 years; mean age: 24.7 [±17.8] years; mean body mass index [BMI]: 24.47 [±8.70] kg/m^2^; Table S4). All were dosed with TMB‐001 0.05% and 31 patients completed the trial. 27 patients could be included in the PK analysis set (excluding patients with major protocol deviations related to drug administration): 13 adults (aged ≥17 years), 6 adolescents (aged 12–16 years) and 8 children aged 4–11 years. The oral‐dose trial enrolled 15 healthy males (age range: 25–54 years; mean age: 42.3 [±9.5] years; mean BMI: 26.35 [±2.31] kg/m^2^); all received isotretinoin orally.

**FIGURE 1:**
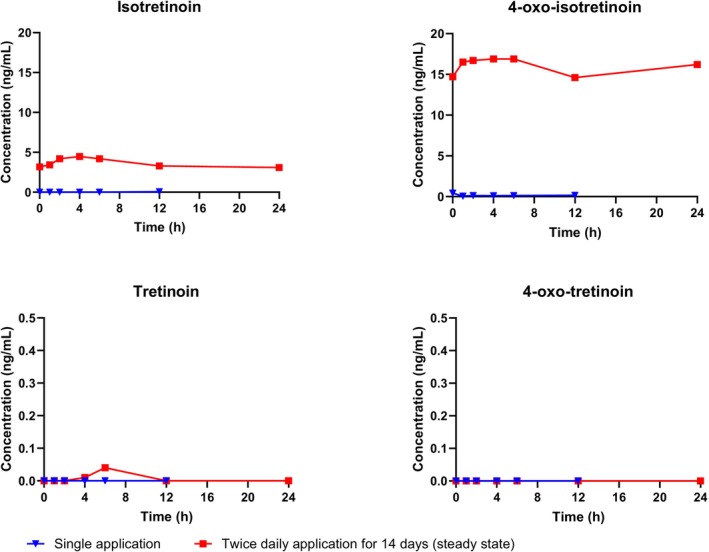
Mean plasma concentration profiles for isotretinoin and its metabolites in adult patients with moderate‐to‐severe congenital ichthyosis after topical application of TMB‐001 0.05% under maximal‐use conditions. Plasma concentrations are plotted *vs.* nominal time and are baseline‐adjusted, that is, obtained by subtracting patient‐specific baseline values for each metabolite at pre‐dose from the concentration at each timepoint. Number of patients in analysis set: 13 adults; number of patients with calculable data: 10 adults.

In ASCEND‐MUsT, a total of 208 plasma samples were analysed (147 samples from adults, 34 from adolescents and 27 from children). Exposure levels for isotretinoin and its geometric isomer and metabolites after topical application of TMB‐001 0.05% were low (Figure 1 and Table 1). The highest steady‐state exposure was seen for 4‐oxo‐isotretinoin, consistent with this being the major metabolite of isotretinoin; the major metabolic pathway of isotretinoin appears to be its oxidation to 4‐oxo‐isotretinoin, catalysed primarily by cytochrome P450 (CYP) isoform 3A4, with further glucuronidation of 4‐oxo‐isotretinoin.^7^ In children aged 6–11 years, the highest mean plasma concentration during steady state was 2.82 ng/mL for isotretinoin and 9.53 ng/mL for 4‐oxo‐isotretinoin, that is, lower than the mean maximum plasma concentration (C_max_) in patients aged ≥12 years. Most PK parameters were not calculable in children, owing to their sparse blood sampling schedule.

**TABLE 1:** Pharmacokinetic parameters for isotretinoin and its metabolites in adult and adolescent patients with moderate‐to‐severe congenital ichthyosis after topical application of TMB‐001 0.05% under maximal‐use conditions for 14 days.

Compound	Age group	Mean AUC_0–24_ (h × ng/mL)	Mean C_max_ (ng/mL)	Median T_max_ (h)
Isotretinoin	Adults (≥17 years)	78.44	4.13	1.50
	Adolescents (12–16 years)	101.34	7.70	14.00
4‐Oxo‐isotretinoin	Adults (≥17 years)	352.95	16.70	5.00
	Adolescents (12–16 years)	432.96	25.44	18.00
Tretinoin	Adults (≥17 years)	0.17	0.04	0.00
	Adolescents (12–16 years)	13.74	4.52	2.00
4‐Oxo‐tretinoin	Adults (≥17 years)	0.00	0.00	0.00
	Adolescents (12–16 years)	10.15	3.60	1.00

*Note*: Number of patients in analysis set: 13 adults and 6 adolescents; number of patients with calculable data: 10 adults and 2 adolescents.

Abbreviations: AUC_0–24_, area under the plasma concentration–time curve from 0–24 h post‐dose; C_max_, maximum plasma concentration; T_max_, time to reach maximum plasma concentration.

In the oral‐dose trial, a total of 313 plasma samples were analysed. The mean C_max_ for isotretinoin was 1160 ng/mL and the mean area under the plasma concentration–time curve from 0–24 h post‐dose (AUC_0–24_) was 10 800 h × ng/mL. Hence, systemic exposure after multiple topical applications of TMB‐001 0.05% proved to be much lower than the exposure after a single oral dose of 80‐mg isotretinoin: mean C_max_ was 280‐fold lower in adults and 150‐fold lower in adolescents; mean AUC_0–24_ was 138‐fold lower in adults and 107‐fold lower in adolescents.

**FIGURE 2:**
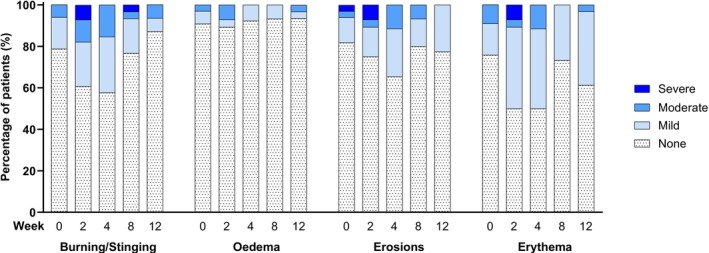
Local skin reactions in patients with moderate‐to‐severe congenital ichthyosis after topical application of TMB‐001 0.05% under maximal‐use conditions. Number of patients in analysis set: 34; number of patients with available data: 33.

Eighty‐six AEs, all non‐serious, were reported for 24 (70.6%) of the patients dosed with TMB‐001 0.05% for up to 12 weeks in ASCEND‐MUsT. Seventy‐six AEs were mild or moderate and 10 were severe (8 administration site reactions, 2 pruritus events); 4 patients permanently discontinued treatment because of AEs, all considered probably treatment‐related by the investigator: 1 adult with severe pruritus, 1 adolescent with 3 events of mild or moderate administration site reactions, 1 adolescent with moderate application site pain and moderate skin irritation and 1 child with severe application site erythema and severe application site dermatitis along with mild chills and mild hidrosis. In addition, 5 patients had their treatment interrupted from 2 to 12 days because of AEs considered probably or possibly treatment‐related by the investigator: 1 adult with severe pruritus, 3 adults with administration site reactions of mild, moderate or unknown severity, and 1 adolescent with mild administration site reactions. There were no AEs consistent with systemic isotretinoin exposure. The most frequently reported AE type was administration site reactions (53 in 18 [52.9%] patients). LSRs occurred in the majority of patients, mainly as erythema (70.6% of patients), burning/stinging (55.9% of patients) and erosions (44.1% of patients); few patients had oedema (17.7%), and none had suspected allergic contact dermatitis. LSRs were reported most frequently at Week 2 and Week 4 and were usually mild (Figure 2).

At Week 12, 55.9% of the patients in ASCEND‐MUsT had a ≥2‐point improvement from baseline on the 5‐point IGA scale (combined scaling and fissuring). The single‐arm design of ASCEND‐MUsT, however, precludes any conclusion on the efficacy of topical isotretinoin for treatment of CI.

In summary, topical application of the isotretinoin ointment formulation TMB‐001 0.05% for 14 days under maximal‐use conditions in patients with CI provided C_max_ and AUC_0–24_ values < 1% of those resulting from a single oral dose of 80 mg isotretinoin. The sample sizes of the trial populations and age groups contributing with data to the analysis were small. Nevertheless, these results from the final analysis of ASCEND‐MUsT add to the sparse data published to date on the PK of topical isotretinoin application. Although a majority of patients had local safety or tolerability issues, most of these were mild. Overall, treatment with TMB‐001 0.05% in this trial was well tolerated, with a safety profile similar to that observed in previous studies.^5^, ^8^ The results reported herein may inform the potential use or further investigation of isotretinoin formulations for topical treatment of various skin conditions.

## AUTHOR CONTRIBUTIONS

Conceptualization: A.M.M., C.G.B., J.M.C.T.; Data curation: A.M.M., C.G.B., H.S., J.M.C.T., J.S.; Formal analysis: A.M.M., C.G.B., H.S., J.M.C.T., J.S., K.H., L.W.L., N.P., S.K., T.N.H., U.B.P.; Investigation: A.M.M., C.G.B., H.S., J.M.C.T., K.H., L.W.L., N.P., S.K., T.N.H., U.B.P.; Methodology: A.M.M., C.G.B., H.S., J.M.C.T.; Writing—review and editing: A.M.M., C.G.B., H.S., J.M.C.T., J.S., K.H., L.W.L., N.P., S.K., T.N.H., U.B.P.

## CONFLICT OF INTEREST STATEMENT

H. S. has served as consultant for Krystal Biotech, Novartis, Roche and SRE GmbH; and as investigator and consultant for Edimer Pharmaceuticals, Pervormance International GmbH, Pierre Fabre and Wyeth Pharma GmbH. C. G. B. has served as investigator for AbbVie, Almirall, Apogee, Daiichi Sankyo, LEO Pharma, Ortho Dermatologics, Sun Pharma, Takeda, Timber and Palvella; and as consultant for AbbVie, Almirall, Amgen, Apogee, Arcutis, Botanix, Connect Biopharma, Dermavent, Eli Lilly, EPI Health/Novan, Incyte, LEO Pharma, Novartis, Ortho Dermatologics, Pfizer, Regeneron Pharmaceuticals, Sanofi, Sun Pharma, Takeda, Teladoc, Triveni and UCB. K. H. has served as investigator for AbbVie, Eli Lilly, Sanofi, Amryt Pharma, Bayer, Cassiopea, Concert Pharma (Sun Pharma), Novartis, Legacy Healthcare and LEO Pharma. U. B. P. has served as advisor, consultant, and speaker for AbbVie, Boots Healthcare, CeraVe, Eli Lilly, Galderma Laboratorium GmbH, Pfizer, Pierre Fabre, Regeneron Pharmaceuticals, Sanofi and Vichy; and as investigator for Amryt Pharma, Bayer, Cassiopea, Concert Pharma (Sun Pharma), Novartis, Legacy Healthcare and LEO Pharma. J. M. C. T. has served as consultant for Abeona Therapeutics, AFT Pharmaceuticals, Amryt Pharma, BridgeBio, Krystal Biotech, Menlo Therapeutics and Nobelpharma; and as investigator and consultant for Castle Creek Biosciences, LEO Pharma, Novartis, Palvella Therapeutics, Pfizer, Regeneron Pharmaceuticals and Timber Pharmaceuticals. A. M. M. was an employee of Timber Pharmaceuticals, a LEO Pharma company, at the time of this trial. J. S. is an employee of LEO Pharma. L. W. L. is an advisory board member for Castle Creek Biosciences, Chiesi, Eli Lilly, Pfizer, Verrica, Regeneron Pharmaceuticals Inc. and Twi Biotechnology; a consultant for AbbVie, Apogee, Chiesi, Kimberly‐Clark, Krystal Biotech, Eli Lilly and Novartis; an investigator with AbbVie, Amgen, Amryt Pharma, Arcutis Biotherapeutics, Apogee, Boehringer Ingelheim, Castle Creek Biosciences, Celgene, Eli Lilly, Galderma, Incyte, LEO Pharma, Mayne Pharma, Novartis, Pfizer, Regeneron Pharmaceuticals Inc., Sanofi, Target Pharma, Trevi Therapeutics, Twi Biotechnology and UCB; and a speaker for Sanofi, Chiesi and Krystal Biotech and participates on data safety monitoring boards for BMS. The remaining authors state no conflict of interest.

## PRINCIPAL INVESTIGATOR STATEMENT

The authors confirm that the PI for this paper is Holm Schneider, MD, and that he had direct clinical responsibility for patients.

## TRIAL REGISTRATION

ClinicalTrials.gov: NCT05295732.

## Supporting information


**Table S1.** Blood sampling schedule in patients with moderate‐to‐severe congenital ichthyosis (ASCEND trial).
**Table S2.** Blood sampling schedule in healthy males (TMB01–101 trial).
**Table S3.** Investigator's Global Assessment 5‐point scale.
**Table S4.** Patient demographics (ASCEND trial).

## Data Availability

De‐identified individual participant data can be made available to researchers via a secured file transfer protocol upon reasonable request. The data are not publicly available due to privacy or ethical restrictions.
