# Supplementary material for: Maximal use of 0.05% topical isotretinoin in patients with congenital ichthyosis results in low systemic exposure
Source: Br J Clin Pharmacol. 2025 Dec 17;92(4):1256–60. doi: 10.1002/bcp.70423 (PMC13021285; doi:10.1002/bcp.70423)
Supplement: Supplementary file 1 — Table S1. Blood sampling schedule in patients with moderate‐to‐severe congenital ichthyosis (ASCEND trial). Table S2. Blood sampling schedule in healthy males (TMB01–101 trial). Table S3. Investigator's Global Assessment 5‐point scale. Table S4. Patient demographics (ASCEND trial). [file BCP-92-1256-s001.docx]

**Maximal use of 0.05% topical isotretinoin in patients with congenital ichthyosis results in low systemic exposure**

Holm Schneider^1^, Christopher G Bunick^2^, Kathrin Hillmann^3^, Thy N. Huynh^4^, Steven Kempers^5^, Nicolai Peschel^1^, Ulrike Blume-Peytavi^3^, Joyce MC Teng^6^, Alan M Mendelsohn^7^, John Stinson^8^, Lara Wine Lee^9^

^1^Department of Pediatrics, University Hospital Erlangen, Erlangen, Germany, ^2^Department of Dermatology, Yale School of Medicine, New Haven, Connecticut, USA, ^3^Department of Dermatology, Venereology and Allergology, Charité Universitätsmedizin Berlin, Berlin, Germany, ^4^Department of Dermatology, University of Mississippi, Jackson, Mississippi, USA, ^5^Associated Skin Care Specialists, New Brighton, Minnesota, USA, ^6^Department of Dermatology, Stanford University School of Medicine, Palo Alto, California, USA, ^7^Timber Pharmaceuticals, a LEO Pharma company, Madison, New Jersey, USA, ^8^LEO Pharma A/S, Ballerup, Denmark, ^9^Department of Dermatology, Medical University of South Carolina, Charleston, South Carolina, USA

**Correspondence:** Holm Schneider, MD. Email: [holm.schneider@uk-erlangen.de](mailto:holm.schneider@uk-erlangen.de)

**Table S1. Blood sampling schedule in patients with moderate-to-severe congenital ichthyosis (ASCEND trial)**

| Day no. | Patient no. | Sampling time points in patients aged ≥12 years |
| --- | --- | --- |
| 1 | All patients | Pre-dose (within 1 hour before morning dose); 1, 2, 4, and 6 hours post-dose; within 1 hour before second daily dose |
| 12 |  | Pre-dose (within 1 hour before morning dose) |
| 14 |  | Pre-dose (within 1 hour before morning dose); 1, 2, 4, 6, and 12 hours post-dose |
| 15 |  | Pre-dose (approximately 24 hours after dose on Day 14) |
| 84 |  | Follow-up (±7 days of Day 84) |
|  | | **Sampling time points in patients aged 6–11 years** |
| 1 and 14 | 1 | Pre-dose (within 1 hour before morning dose); 1 hour post-dose |
| 1 and 14 | 2 | Pre-dose (within 1 hour before morning dose); 2 hours post-dose |
| 1 and 14 | 3 | Pre-dose (within 1 hour before morning dose); 4 hours post-dose |
| 1 and 14 | 4 | Pre-dose (within 1 hour before morning dose); 6 hours post-dose |
| 1 and 14 | 5 | Pre-dose (within 1 hour before morning dose); 8 hours post-dose |
| 1 | 6 | Pre-dose (within 1 hour before morning dose); 24 hours post-dose |
| 1 and 14 | 7 | Pre-dose (within 1 hour before morning dose); 12 hours post-dose |
| 1 and 14 | 8 | Pre-dose (within 1 hour before morning dose); 4 hours post-dose |
| 1 and 14 | 9 | Pre-dose (within 1 hour before morning dose); 24 hours post-dose (Day 14 only) |

All sampling times were relative to morning dose.

**Table S2. Blood sampling schedule in healthy males (TMB01-101 trial)**

| Day no. | Sampling time points |
| --- | --- |
| 1 | Pre-dose; 0.5, 1.0, 1.5, 2.0, 2.5, 3.0, 3.5, 4.0, 6.0, 8.0, 10.0, 12.0, 14.0, 16.0 hours post-dose |
| 2 | 24.0 and 36.0 hours post-dose |
| 3 | 48.0 hours post-dose |
| 4 | 72.0 hours post-dose |
| 5 | 96.0 hours post-dose |
| 6 | 120.0 hours post-dose |

All sampling times were relative to the single dose given on Day 1.

**Table S3. Investigator’s Global Assessment 5-point scale**

| Score | Category | Combined scaling and fissuring scores |
| --- | --- | --- |
| 0 | Clear | No scaling and no roughness, no fissuring. |
| 1 | Almost clear | Occasional fine scales, hardly palpable roughness (mostly smooth), rare fissuring and/or clinically insignificant fissuring severity. |
| 2 | Mild | Small and fine scales predominate, no more than a few large scales, mild roughness on palpation, few fissures with minimal pain or symptoms that impair patient ADL and/or minimally clinically significant fissuring severity. |
| 3 | Moderate | Large rather thick scales predominate, coarse roughness on palpation, multiple fissures with possible pain or symptoms that somewhat impair ADLs and/or clinically significant fissuring severity. |
| 4 | Severe | Large coalescent scales dominate, sharp edges on palpation with plate-like hyperkeratosis, numerous fissures and/or pain and symptoms that impair ADLs and/or very clinically significant fissuring severity. |

ADL = activities of daily living.

**Table S4. Patient demographics (ASCEND trial)**

| Category | N=34 |
| --- | --- |
| **Age at baseline, years** | |
| Mean (SD) | 24.7 (17.88) |
| Median (min, max) | 18.0 (4, 64) |
| **Age group, n (%)** | |
| 6–11 years^a)^ | 9 (26.5%) |
| 12–16 years | 7 (20.6%) |
| ≥17 years | 18 (52.9%) |
| **Body mass index, kg/m^2^** | |
| Mean (SD) | 24.47 (8.696) |
| Median (min, max) | 22.60 (13.20, 51.60) |
| **Sex, n (%)** | |
| Male | 18 (52.9%) |
| Female | 16 (47.1%) |
| **Race, n (%)** | |
| White | 21 (61.8%) |
| Black or African American | 7 (20.6%) |
| Asian | 3 (8.8%) |
| American Indian or Alaska Native | 1 (2.9%) |
| Other | 1 (2.9%) |
| Not Answered | 1 (2.9%) |
| **Ethnicity, n (%)** | |
| Hispanic or Latino | 2 (5.9%) |
| Not Hispanic or Latino | 25 (73.5%) |
| Unknown/Missing | 7 (20.6%) |
| **Congenital ichthyosis subtype, n (%)** | |
| Autosomal recessive congenital ichthyosis (ARCI) | 26 (76.5) |
| Recessive X-linked ichthyosis (RXLI) | 8 (23.5) |

a) The age group ‘6–11 years’ includes one patient who was 4 years old when entering the trial (upon sponsor approval). N = Number of patients enrolled; n = number of patients in category; SD = standard deviation.
